# The prognostic impact of human papillomavirus status following treatment failure in oropharyngeal cancer

**DOI:** 10.1371/journal.pone.0181108

**Published:** 2017-07-21

**Authors:** Eesha Dave, Umut Ozbek, Vishal Gupta, Eric Genden, Brett Miles, Marita Teng, Marshall Posner, Krzysztof Misiukiewicz, Richard L. Bakst

**Affiliations:** 1 Department of Medical Education, Icahn School of Medicine, New York, New York, United States of America; 2 Department of Population Science and Policy, Mount Sinai Hospital, New York, New York, United States of America; 3 Department of Radiation Oncology, Icahn School of Medicine at Mount Sinai, New York, New York, United States of America; 4 Department of Otolaryngology-Head and Neck Surgery, Icahn School of Medicine at Mount Sinai, New York, New York, United States of America; 5 Department of Hematology-Oncology-Tisch Cancer Institute, Icahn School of Medicine at Mount Sinai, New York, New York, United States of America; Taipei Medical University, TAIWAN

## Abstract

**Introduction:**

Despite the human papillomavirus conferring a better prognosis in the primary treatment setting, the prognostic impact of viral status after treatment failure in oropharyngeal squamous cell carcinoma patients is poorly understood.

**Methods:**

We retrospectively identified 33 oropharyngeal squamous cell carcinoma (OPC) patients with local and/or distant disease recurrence post-treatment, and looked at metastatic patterns, time to failure and survival patterns by HPV status.

**Results:**

Median overall survival following local failure was not significantly different by HPV status (17 months for HPV+ vs. 14 months for HPV-, p = 0.23). However, following distant failures, HPV+ patients lived significantly longer than HPV- patients (median 42 months vs. 11 months, p = 0.004). HPV- patients were more likely to have locoregional failures as compared to HPV+ patients (p = 0.005), but the difference in distant failure between both groups was not significant (p = 0.09). HPV+ patients were more likely to develop metastases to sites other than the lung and bones.

**Conclusion:**

HPV positivity predicts a favorable prognosis with the potential for long-term survival following distant, not locoregional, failures. These results have important implications for the aggressiveness of treatment and type of surveillance imaging performed.

## Introduction

Among patients with newly diagnosed oropharyngeal squamous cell carcinoma (OPC), those with human papillomavirus positive (HPV+) disease have a more favorable disease course and prognosis than those with HPV negative (HPV-) disease. Studies have proven that patients with HPV+ OPC have better local regional control and a longer overall survival and progression-free survival compared to HPV- OPC patients after curative therapy [1–5]. However, despite HPV positivity conferring an enhanced prognosis, there is a small subset of patients who fail definitive treatment [5–6].

In addition, previous research suggests that after definitive therapy, HPV positive patients may have a tendency to experience more unique patterns of soft tissue and visceral metastases [5–7]. They may also experience distant metastases at later intervals than HPV- patients [5–6]. However, it remains unknown as to whether HPV positivity continues to convey a survival advantage and dictate prognosis following distant and/or local failure. To investigate this question, we identified OPC patients who failed definitive therapy, and analyzed their survival and patterns of metastasis following treatment failure to determine if HPV status still drives prognosis.

## Materials & methods

### Study population

We conducted an Institutional Review Board approved retrospective departmental chart review. All data was fully anonymized before access by the researchers, and the IRB or ethics committee waive the requirement for informed consent. We identified 33 patients treated between 2006–2015 with OPC, who had experienced local and/or distant failures after definitive therapy. We analyzed their demographics, clinical information, treatment type, and failure patterns. Failures were defined as either a local recurrence and/or distant metastasis of the disease after definitive therapy as determined by imaging and pathology. Persistent disease was defined as evidence of disease on imaging after finishing the prescribed course of treatment. Imaging studies used to determine distant metastasis included positron emission tomography (PET) or computerized tomography (CT).

Specifically, we collected information including gender, age, HPV status, HPV serotype when available, disease stage, primary site, radiation dose, radiation volume, treatment course, smoking status, alcohol use, salvage therapy modalities used, date of failure, type of failure, date of last follow-up, sites of metastases, any subsequent episodes of treatment (radiation, surgery, chemotherapy) and current disease status (disease free versus not). DNA polymerase chain reaction and/or p16 immunostaining were used to identify the HPV status of the entire cohort. All patients identified either underwent definitive chemoradiation therapy (chemoRT) or surgery followed by adjuvant therapy and were without evidence of disease following their respective definitive treatment. Patients with persistent disease following definitive treatment were excluded.

### Definitive treatment at time of initial diagnosis

All individuals received RT in this study and were treated with intensity-modulated radiation therapy. Definitive treatment in this cohort consisted of either definitive chemo/RT or surgical resection with adjuvant radiotherapy or chemoradiotherapy based on prognostic factors. HPV status was not taken into account when planning definitive treatment at time of diagnosis. Radiation fields and dosages, along with chemotherapy agents and surgical procedures, were similar regardless of HPV status. The treatments they received are outlined below, and are detailed in Table 1.

**Table 1:** Patient characteristics and demographics.

Parameter	HPV+, n = 20	HPV-, n = 13	P-value
**Gender**			0.052
Male	20	10	
Female	0	3	
**Age (median, range)**	56 (37–82)	59 (39–73)	0.382
**Alive (%)**	8 (40%)	2 (15%)	0.246
**HPV+ Type**			
Unknown	8		
16	10	-	
33	1	-	
35	1	-	
**Primary Site**			0.063
Tonsil	11	2	
Base of Tongue	7	8	
Non-specified	2	3	
**Tobacco Use**			0.073
<20 pack-years	12	3	
= />20 pack-years	8	10	
**Alcohol Use**			0.139
None/social	16	7	
Heavy use	4	6	
**Staging (AJCC**^a^**)**			0.666
I	0	0	
II	2	0	
III	4	4	
IVA	11	6	
IVB	3	3	
IVC	0	0	
**Radiation Therapy (initial)**			
Dose (Gy (median, min-max)	7000 (7200–5600)	7000 (6000–7200)	0.092
**Chemotherapy**			
Induction	7	7	0.472
Concurrent	18	11	1.0
**Primary Surgery (total)**	9	3	
**Median time from RT completion to failure (months)**	8.6	4.0	

^a^American Joint Committee on Cancer

All patients in the surgical cohort received transoral robotic surgery (TORS) or traditional oropharyngeal resection including unilateral or when appropriate bilateral neck dissections. In the surgical cohort, 42% of patients received TORS. Patients undergoing surgery received radiation based on standard National Comprehensive Cancer Network guidelines for intermediate- and high-risk feature [8]. All patients in this group had a gross total resection. Microscopic margins were positive in 2 patients and extracapsular extension was present in 3 patients. All patients but one in the cohort completed their assigned courses of radiation therapy. This single patient who did not complete RT developed a pulmonary embolism during treatment, but had no evidence of disease at the time of radiation termination.

### Analysis

Categorical variables were compared using Chi-square or Fisher’s exact tests. Continuous variables were compared using Mann-Whitney U test. Time to distant and local failures were characterized with the use of Kaplan-Meier plots and log-rank tests were used to compare the outcomes between HPV positive and negative patients. All reported P-values are two-sided, and P-values <0.05 were considered significant. All analyses were performed in SAS v9.4 statistical software (SAS Institute, Cary, NC, USA).

## Results

### Patient characteristics

The median age of the patients was 58.8 years. The majority of patients were male (91%), and the HPV+ group was exclusively male. In patients where DNA PCR data was available HPV 16 was the most common genotype. The tonsil was the most common primary subsite for the HPV+ group, and base of tongue was the most common primary subsite for the HPV- group. The demographic and clinical characteristics of the cohort can be found in Table 1. At initial diagnosis, all patients underwent extensive clinical and radiographic staging with PET/CT and had no evidence of metastatic disease. At definitive treatment completion, patients had no evidence of disease by definition of entry criteria.

### Patterns of failure

Time to failure from completion of definitive treatment occurred at a median of 8.6 months (range 1.7–26.3 months) for the HPV+ group, and a median of 4.0 months (range 1.4 to 22.0 months) for the HPV- group. This value was 5.7 months for the entire cohort. Failures were categorized as locoregional only, distant only and locoregional and distant. A breakdown of types of failure by HPV status is listed in Tables 2 and 3. Median follow-up time for the cohort since the time of initial failure was 10.8 months.

**Table 2:** Summary of type of failure by HPV status.

Failure Type	HPV+ (n = 20)	HPV- (n = 13)
Locoregional only	5 (25%)	10 (77%)
Distant only	11 (55%)	3 (23%)
Locoregional & Distant simultaneous	4 (20%)	0
Total LR^a^	9 (45%)	10 (77%)
Total DM^b^	15 (75%)	3 (23%)

^a^Locoregional

^b^Distant metastases

**Table 3:** Type of failure by stage and HPV status.

	HPV+			HPV-		
Stage	Locoregional	Distant	Both	Locoregional	Distant	Both
**I**	0	0	0	0	0	0
**II**	1	1	0	0	0	0
**III**	0	4	0	4	0	0
**IVA**	3	5	3	4	2	0
**IVB**	1	1	1	2	1	0

### Local failures

Of the 15 patients (10 HPV- and 5 HPV+) who experienced a local failure, 93% had advanced stage disease (stage III or IV). In the HPV+ group, 40% had a significant history of smoking (40+ packs/year) and 20% had a significant alcohol use history. In the HPV- group, 40% had a significant history of smoking, and 40% had a significant alcohol use history.

Of note, HPV- patients have more locoregional failures compared to HPV+ patients statistically (p-value = 0.005). However, the difference in distant failure between HPV groups is not statistically significant (p-value = 0.09).

### Distant metastases

**Fig 1:**
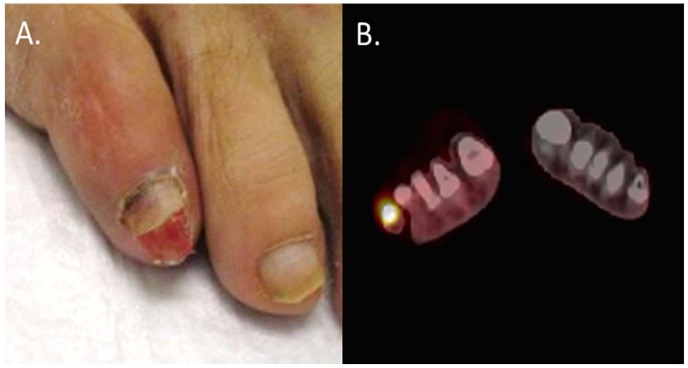
(A) Photograph of distant metastasis to right fifth toe. (B) Pet scan of metastasis to right fifth toe.

A summary of sites of distant metastases by HPV status is available in Table 4. 75% of HPV+ patients and 23% of HPV- patients had distant metastases. PET/CT scans indicated sites of distant metastases including the lungs, lymph nodes and bone. Both HPV+ and HPV- patients experienced metastases to visceral organs. The HPV+ group had more diversified sites of metastases including extremities and soft tissues (Fig 1A and 1B) and the brain. Of the distant failures, 33% of HPV- and 20% of HPV+ patients had oligometastatic disease. In the HPV+ cohort, 10% of patients experienced distant metastases distal to the thigh.

**Table 4:** Distant metastases sites by HPV status.

Distant Metastasis Site	HPV+ Group	HPV- Group
Lung	12	3
Lymph Nodes below the clavicle	8	3
Bone	4	1
Visceral organs	5	2
Extremity/soft tissue	2	0
Brain	1	0

### Treatment following initial failure

For patients who developed distant failures, 86% received systemic therapy, and in addition, 50% received surgery. Of these surgeries, 71% were lung resections. For patients who failed locally only, 40% received salvage surgery and 27% received salvage RT. For a more complete breakdown of post-curative treatments for the patients who experienced distant and local failures by HPV status, see Table 5. There were a limited number of patients with such poor performance status at time of disease failure that palliative care was appropriate.

**Table 5:** Treatment after failure by failure type and HPV status.

**Distant Failures**		
Treatment	HPV+ (%) (n = 10)	HPV- (%) (n = 3)
Surgery, Radiation, Chemo	30	0
Surgery, chemo	20	33
Radiation, chemo	20	0
Chemo only	20	67
Surgery only	10	0
**Local Failures**		
Treatment	HPV+ (%) (n = 5)	HPV- (%) (n = 5)
Surgery, Radiation, Chemo	20	20
Surgery, chemo	20	20
Radiation, chemo	40	0
Chemo only	0	40
Surgery only	20	20
**Synchronous**		
Treatment	HPV+ (%) (n = 3)	HPV- (%) (n = 0)
Surgery, Radiation, Chemo	0	0
Surgery, chemo	67	0
Radiation, chemo	0	0
Chemo only	33	0
Surgery only	0	0

### Survival analysis by failure type and therapy

**Fig 2:**
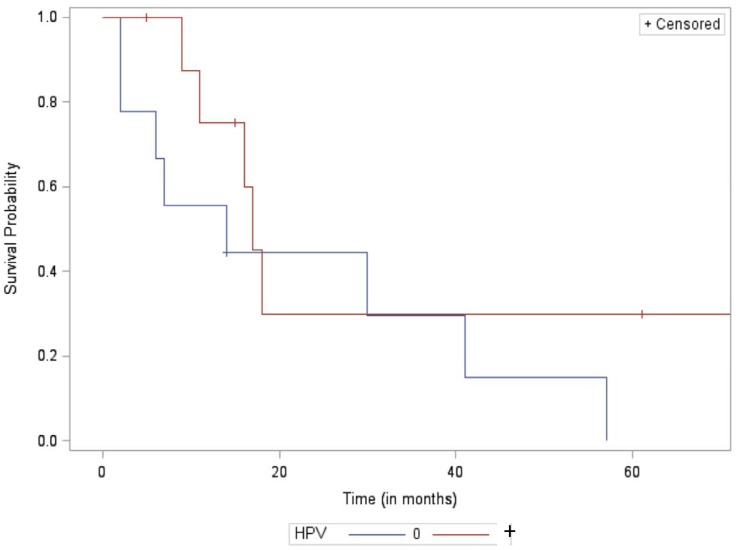
Kaplan-Meier survival curve of the local failure cohort.

**Fig 3:**
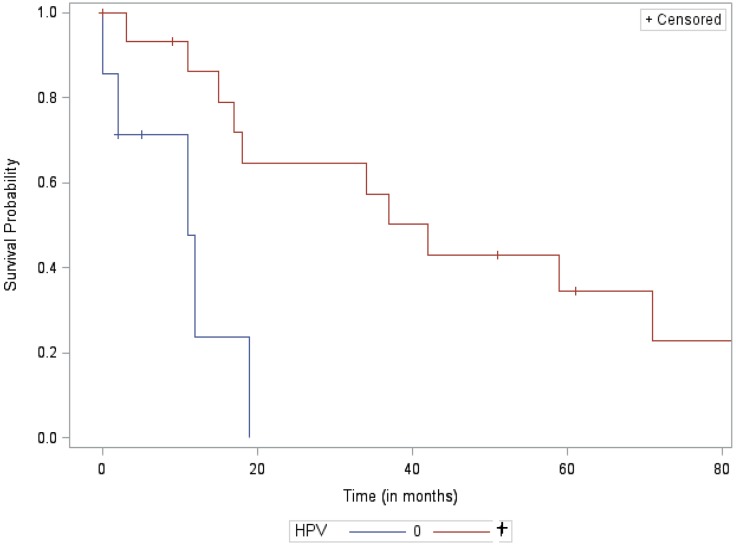
Kaplan-Meier curve for the distant failure cohort.

Among patients with locoregional failure, median survival from treatment failure did not significantly differ between the HPV positive and HPV negative groups at 17 months and 14 months, respectively (p = 0.23). However, among patients who failed distantly, median survival from treatment failure was significantly longer for HPV+ than HPV- patients at 42 months and 11 months, respectively (p = 0.004). Furthermore, at the 2-year mark, survival rates for patients after distant failure were 65% for HPV+ vs. 0% for HPV- patients. Kaplan-Meier curves of survival analysis are included below in Figs 2 and 3.

## Discussion

This study attempts to address the prognostic value of HPV status following treatment failure. This data is derived primarily from definitive radiation-based treatment, and assumed to be true for primary surgery followed by appropriate adjuvant therapy as well [1,9]. In general, HPV+ patients have better survival rates and prognoses than HPV- patients [10]. However, HPV+ patients who do happen to experience a recurrence, experience metastases after longer intervals of time, and these metastases occur in more diverse locations within the body [4,6].

In our study, HPV positivity appears to influence survival following distal failure only. By contrast, prognosis following local failure does not seem to be as driven by viral positivity. The mechanism by which HPV continues to convey a better prognosis following distant failure remains unclear. A number of factors could be involved including increased sensitivity to chemotherapy and radiotherapy, generally fewer comorbidities, and a more limited tobacco and alcohol exposure history [4]. Decreased survival after locoregional recurrence may be due to acquired resistance to radiotherapy and chemotherapy with clonal selection of resistant cells resulting in infield recurrences, which has been cited in literature about HPV-associated anal cancers[11]. This does not seem to extend to distant metastases in those who receive CRT.

Thus, in HPV+ patients, long term survival following distant metastases was substantial in comparison to HPV- patients. Historical results in clinical trials where the majority of patients with recurrent/metastatic disease are HPV- also support this finding [12]. In light of the obvious responsiveness of the recurrent disease among HPV+ patients with distant failure, more aggressive therapy may be considered depending on the patients’ functional status. Given that the HPV- cohort has a uniformly poor prognosis, the need for more aggressive therapy merits further investigation.

In our study, local failure irrespective of HPV status portended a very poor prognosis. The feasibility of salvage surgery or radiation, acquired or intrinsic resistance to radiotherapy, and the evolution of the cancer likely drive survival following local failure rather than the viral status. However, in the era of treatment de-intensification, the poor prognosis associated with local failures even in the HPV+ patients should raise caution about the ease of salvaging them should reduced radiotherapy or CRT result in local failure. It is also important to note that a distant failure following chemo/RT does suggest that the disease is radio-resistant or chemotherapy resistant.

While our data showed that HPV+ patients were more likely to have distant metastases and HPV- patients were more likely to fail locoregionally, the difference in distant failures was not significant. Regardless, in line with previous studies, we found that metastatic patterns are distinct between HPV+ and HPV- patients [5–6,13]. Metastases to the lungs and lymph nodes below the clavicle were most common in both subpopulations. However, the HPV+ group also experienced metastases to more untraditional sites including the bone, visceral organs, extremity/soft tissue and brain. This has important implications for follow-up imaging if and when performed. In addition to the lungs, one must consider the imaging modality and the necessity of whole body imaging, given the wide possibility of metastatic occurrences. Additionally, a complaint involving the soft tissue or extremities should cause one to include metastasis in the differential. HPV subtype also may also have a role, yet the impact of viral subtype currently remains unknown. Given that serotypes are not factored into treatment, treating all serotypes in the same way may impact the recurrence profiles of the patients, with certain serotypes conferring more aggressive disease courses [7].

This study has several limitations including the relatively small cohort size and retrospective nature of the study that can lead to inherent bias. Additionally, while HPV status was not a factor in determining treatment, HPV+ patients did tend to receive more therapy when they relapsed potentially accounting for some fraction of their improved survival. While HPV subtyping was not available for all patients, it would be important going forward to ascertain how the different biology of non-HPV16 viral subtypes impact prognosis. Lastly, multivariate analysis was not performed given the limited number of deaths in our cohort, but would be worthwhile in future studies. Despite these limitations, this represents one of the largest studies evaluating failure patterns by HPV status in oropharyngeal cancer and significantly contributes to our growing understanding as to how best to treat, counsel and follow patients in the setting of treatment failure.

## Conclusion

Similar to the curative primary treatment setting, HPV positivity continues to drive prognosis following distant failure but does not appear to affect local regional failure after radiotherapy. Notably, in our cohort, HPV negative patients had a predilection to fail locoregionally. HPV+ patients can exhibit a variety of metastatic pattern, with longer periods to recurrence, but have the potential for long-term survival despite these metastases. Collectively, this has implications for imaging and the need for aggressive therapy following failure in HPV+ patients.

## Supporting information

S1 File HPV- patients. (XLSX)

S2 File HPV+ patients. (XLSX)
